# Viewpoint: The Classical Symmetry Number

**DOI:** 10.1021/acs.jpca.6c01212

**Published:** 2026-07-27

**Authors:** David Rovnyak, Henry B. Rovnyak

**Affiliations:** 1 Department of Chemistry, 4517 Bucknell University, 1 Dent Drive, Lewisburg, Pennsylvania 17837, United States; 2 311308 Purdue University, West Lafayette, Indiana 47907, United States

## Abstract

The statistical mechanical description of molecular rotation employs the concept of the symmetry number (σ), which classically is the number of identical configurations of a molecule accessible through rotations (IUPAC; Compendium of Chemical Terminology, fifth ed. International Union of Pure and Applied Chemistry; 2025). However, a meta-analysis of physical chemistry source materials finds that the IUPAC definition is not widely presented, nor is a formal method for determining σ always identified to readers. Materials tend to use informal reasoning or treat σ as self-evident. For example, it is common to justify the symmetry number of methane (CH_4_, σ = 12) by invoking successive *C*_3_ operations around each bond. Such an approach can overcount the initial position or cause confusion by overlooking the *C*_2_ axes and does not constitute a general method for determining σ. This Viewpoint supports wider adoption of the canonical IUPAC definition of σ in physical chemistry disciplinary materials and then reviews three formal methods for determining it in rigid molecules: (i) using the proper rotational subgroup to count all proper rotational symmetry operations (*C*_*n*_, *C*_*n*_^–1^) and the identity (*E*), (ii) using combinatorics to count label-superimposable configurations, and (iii) using a group theoretical method, the Schreier-Sims algorithm. The method used should be identified and presented in sufficient detail to be applied to other rigid molecules. The method of determining the order of the proper rotational subgroup merits greater use since it is most closely associated with the IUPAC standard and is widely accessible.

## Introduction

The symmetry number σ is the number of identical configurations of a molecule accessible through rotations (IUPAC),^1,2^ and is commonly applied to rotational problems in the classical limit and assuming separable rigid rotor and harmonic oscillator wave functions (e.g., RRHO approximation). The IUPAC standard does not prescribe a formal method for determining σ, where three methods to find σ are: (i) the order of the proper rotational subgroup, (ii) combinatorics, or (iii) group theory (Schreier-Sims^3–6^ ). Classically, σ is used to avoid overcounting configurations in the study of rotational entropy, and supports rich physical chemistry, including recent examples in this journal.^5,7–11^ It is challenging to introduce the symmetry number since its foundations in quantum statistical mechanics are nontrivial, where even seemingly simple cases (H_2_, HD, and D_2_) can be difficult to explain. Fortunately, the classical high-temperature definition of the symmetry number in the RRHO framework has many applications. Distorted shapes, thermal inversion,^12^ and internal motions^4,5,13,14^ require using symmetry numbers developed specifically for those cases, particularly internal symmetry numbers.^4,5,15,16^ The classical symmetry number of rigid perfect shapes will be focus of this Viewpoint.

Physical chemistry and statistical mechanics materials often cover molecular rotational partition functions, including the symmetry number (σ) concept. A large sample of print and online instructional materials was consulted.^15,17–47^ These sources largely focused on the classical use of σ, but varied widely in how it was presented. Authors generally favored making the symmetry number of several molecular shapes a learning goal, including in end-of-chapter questions. However, many sources do not give the IUPAC definition and often relied on informal arguments. Even if the IUPAC definition was provided, subsequent examples may have used an informal method, or treated σ as obvious. Some sources appeared to use arguments from group theory, but informally and without identifying or providing exposition on its general use. Other sources solely treat diatomics and introduce the factor of 2 correction to homonuclear rotational partition functions with minimal mention of the symmetry number concept.

Using informal methods for σ risks creating misconceptions and does not equip readers to tackle other molecules. A popular example in both introductory texts and more advanced source books is methane (CH_4_, σ = 12), where they often solely focus on the four *C*_3_ axes to generate σ, but neglect the *C*_2_ axes. In a recent course, it was noticed that the initial position was overcounted this way, and it was questioned why *C*_2_ rotations were used to justify the symmetry number of other molecules, but *C*_2_ axes were ignored in counting σ for methane.

This Viewpoint addresses these findings by examining informal methods first, and then reviewing three formal methods for finding σ (order of proper rotational subgroup, combinatorics, and group theory). Then, while observing that all three methods have merits, this Viewpoint advocates for broader use of the ‘order of proper rotations’ approach since it relates well to the IUPAC definition and is widely accessible.

## Methods

A meta-analysis of the presentation of the symmetry number concept throughout print and online source materials was conducted. In total 32 sources were obtained as a convenience sample of the broader literature on molecular rotational partition functions. Both recent and older texts were included, as well as keystone texts and those intended for both introductory and advanced audiences. The GAP (Groups, Algorithms, Programming) software is publicly available^6^ and was used on a typical personal computer and its use has been described by Gilson and Irikura.^5^ The GAP software was consulted as a part of this work, but is not explicitly the focus of this Viewpoint.

## Results

The meta-analysis conducted here found that some sources may not identify or provide exposition on a formal method to obtain σ, and instead employ a range of informal methods. By ‘informal’, it is meant that σ is counted in a way that does not have a stated theoretical basis, and without guidance to apply it to other molecules. Informal approaches may mix methods or use shortcuts, and may even contain misconceptions. For example, in the surveyed sources, some treated σ as obvious and others relied on nonrotational symmetry elements as crutches, and we recommend against both of those practices.

One specific informal approach to σ is particularly common and is examined here further. It often invokes methane where, to summarize in one generic example, it asserts that one can count all 12 configurations of methane by rotating about all four C-H axes. Some sources may identify these as *C*_3_ axes. It should be recognized that this informal procedure actually overcounts the initial position, which can only be avoided by placing each label at the ‘top’ position for each of the CH rotations,^41^ a step which is not obvious, especially to introductory audiences. Even if sources do take care to avoid overcounting the initial position, readers may confuse this informal method with the IUPAC definition since it draws on some of the proper rotations in the symmetry group but not all. In a recent experience, audiences questioned how this informal approach could be applied more broadly, why the initial configuration is overcounted, and why the *C*_2_ axes were ignored.

A minor consideration is that methods may rely on using successive rotations to count configurations. Elements of symmetry groups are not usually denoted in terms of successive rotations (Table 1, *C*_6_^2^ = *C*_3_ in *D*_6h_), where relying on successive rotations is not usually consistent with symmetry group notation. Finally, we also observed in the survey a very small number of sources using incorrect explanations, but these instances were scarce and viewed as outliers, and are not treated further.

**Table 1:** Selected Molecular Shapes and Their Classical Symmetry Numbers Are Given, Along with the Proper Rotations (and *E*) That Generate the Symmetry Number from a Common Starting Position^a^

**Description**	**Examples**	**Symmetry Number (σ)**	**Point Group**	**Rotational Subgroup** ^a^	**Condensed Schreier- Sims Product**
diatomic (het.)	XY: HCl, NO	1	*C*_∞*v*_	*E*	1·1
diatomic (homo.)	X_2_: N_2_, O_2_	2	*D*_∞*h*_	*E*, *C*_2_	2·1
linear triatomic	YXY: CO_2_	2	*D*_∞*h*_	*E*, *C*_2_	2·1
trigonal planar	XY_3_: BF_3_	6	*D*_3*h*_	*E*, 3*C*_2_, *C*_3_, *C*_3_^–1^	3·2
trigonal pyramid[Table-fn t1fn4]	XY_3_: PCl_3_	3	*C*_3*v*_	*E*, *C*_3_, *C*_3_^–1^	3·1
tetrahedron	XY_4_:CH_4_, PO_4_^3‑^	12	*T*_*d*_	*E*, 3*C*_2_, 4*C*_3_, 4*C*_3_^–1^	4·3
square planar	XY_4_: XeF_4_	8	*D*_*4* *h*_	*E, C*_4_, *C*_4_^–1^, *C*_2_, 2*C*_2_^′^, 2*C*_2_^″^	4·2
planar hexagon	X_6_Y_6_: C_6_H_6_	12	*D*_6*h*_	*E*, *C*_2_, 3*C*_2_^′^, 3*C*_2_^″^, *C*_3_, *C*_3_^–1^, *C*_6_, *C*_6_^–1^	6·2
octahedron	XY_6_: SF_6_	24	*O*_*h*_	*E*, 6*C*_2_, 4*C*_3_, 4*C*_3_^–1^, 6*C*_4_, 3*C*_2_^′^	6·4

a The final column pertains to the group theory method (see text).

b Full symmetry groups are not shown; see Cotton for complete groups.^48^ We distinguish inverses here, but tables may report, 2*C*_3_ = *C*_3_ + *C*_3_^–1^, 8*C*_3_ = 4*C*_3_ + 4*C*_3_^–1^, etc. Inverse elements may be reported as *C*_*n*_^*n*–1^or*C*_*n*_^–1^, where we use the latter.

c If using ammonia as an example, it should be recognized that it undergoes inversion by tunneling as well as thermally at higher temperatures; while the rigid limit is σ = 3, inversion doubles configurations and leads to σ = 6.^5,12^

We next present three distinct formal methods, with accompanying examples and graphics, that can be used for determining σ: the order of proper rotations, combinatorics, and group theory. Each has merits and challenges, where it is recommended that sources should more widely adopt the order of proper rotations method.

## The Order of Proper Rotations Method: Methane E, *C*_3_, *C*_3_^–1^, and *C*_2_ Elements Comprise σ

The classical symmetry number is the total number of identical configurations accessible by proper rotations.^1^ In the context of molecular symmetry, it is the number of elements (order) of the proper rotational subgroup, which includes identity. This ‘order of proper rotations’ method is verified here for methane, an XY_4_ tetrahedron (see Table 1 or 2 for the rotational subgroup of the full *T*_d_ symmetry group). The arbitrary initial configuration (Figure 1 **A**) corresponds to the identity operator, *E*, counting 1 toward σ. Rotations about each of the three *C*_2_ axes applied to the same starting position yield an additional configuration each, adding 3 to σ (Figure 1 **B**). Designating each atom in turn to be fixed enables the four *C*_3_ and four *C*_3_^–1^ rotations to be applied to the initial configuration, generating eight more configurations (Figure 1 **C**). All proper rotations in the symmetry group have contributed, yielding σ = 1 + 3 + 8 = 12. This method of counting the proper rotations of the point group (*T*_*d*_ for methane) is not typically employed in texts, although an example is given by Silbey, Alberty, and Bawendi.^40^

**Figure 1:**
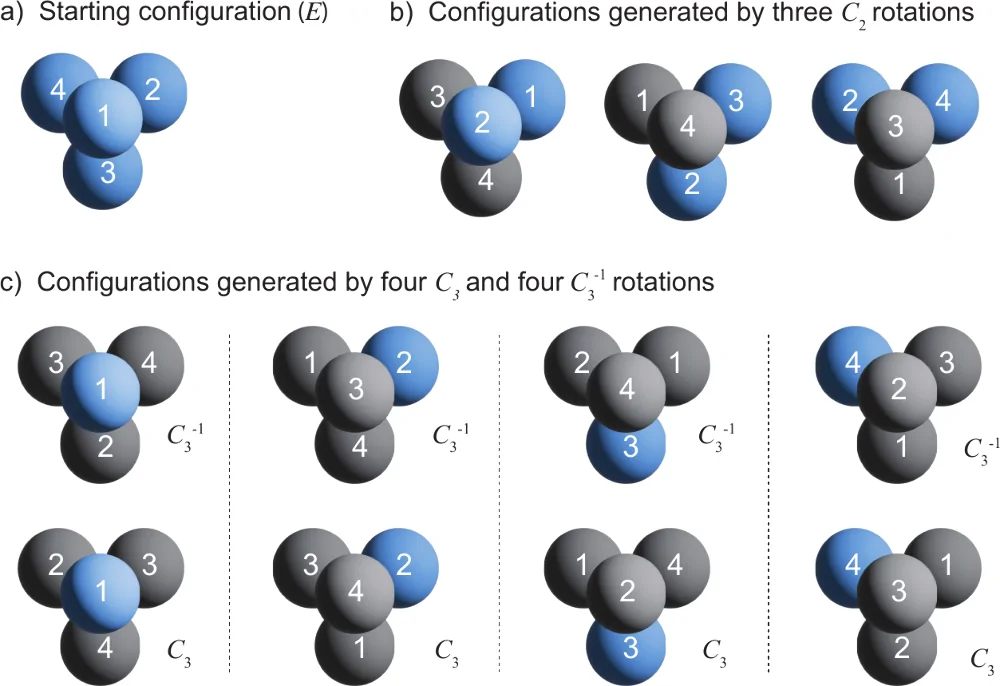
‘Sum of proper rotations’ method for justifying the symmetry number of methane is illustrated. The 12 identical configurations which preserve the relative label order of a perfect XY_4_ tetrahedron (e.g., CH_4_) are generated from one starting configuration with each member of the proper rotational subgroup (see Table 1 or 2): (a) an arbitrary starting label configuration; (b) the three *C*_2_ rotations, and (c) the operation of four *C*_3_ and four *C*_3_^–1^ rotations. In (b), the axis of rotation bisects the blue atoms; in (c), the blue atoms correspond to each axis of rotation.

**Table 2:**
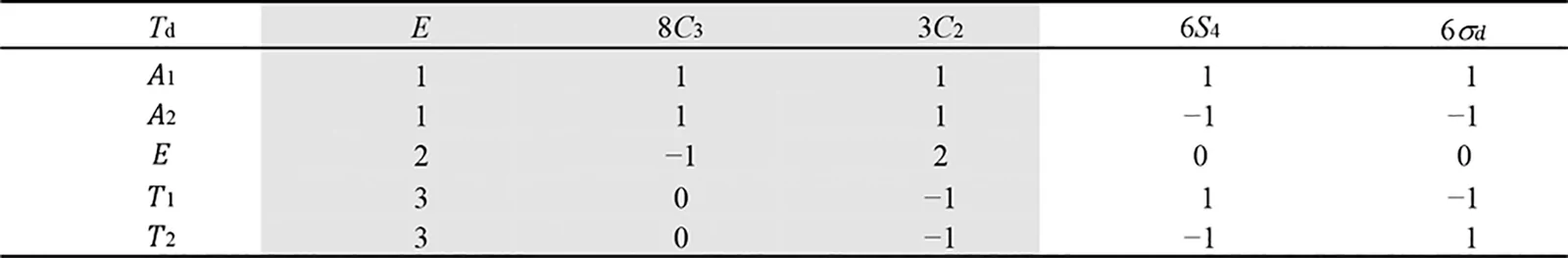
The Character Table of the *T*_d_ Point Group Is Shown for a Perfect Tetrahedron^a^

a It is conventional that the proper rotational subgroup elements (grey shading, including *E*) appear first, enabling a quick assessment of the symmetry number (*E* + 8*C*_3_ + 3*C*_2_ = 12).

The rotational subgroup of the full symmetry group can also be conveniently read from character tables, with the proper rotational subgroup depicted in the first columns, as shown by the shaded region for *T*_*d*_ in Table 2 . A further example is benzene, where σ is verified in Figure 2 by applying each element of the proper rotational subgroup of *D*_6h_ (Table 1) to a common initial configuration. Finding σ as the order of proper rotations via symmetry group tables (Table 1) or character tables (Table 2) is widely applicable and connects closely with the definition of σ, but may rely on some additional background knowledge of molecular symmetry groups.

**Figure 2:**
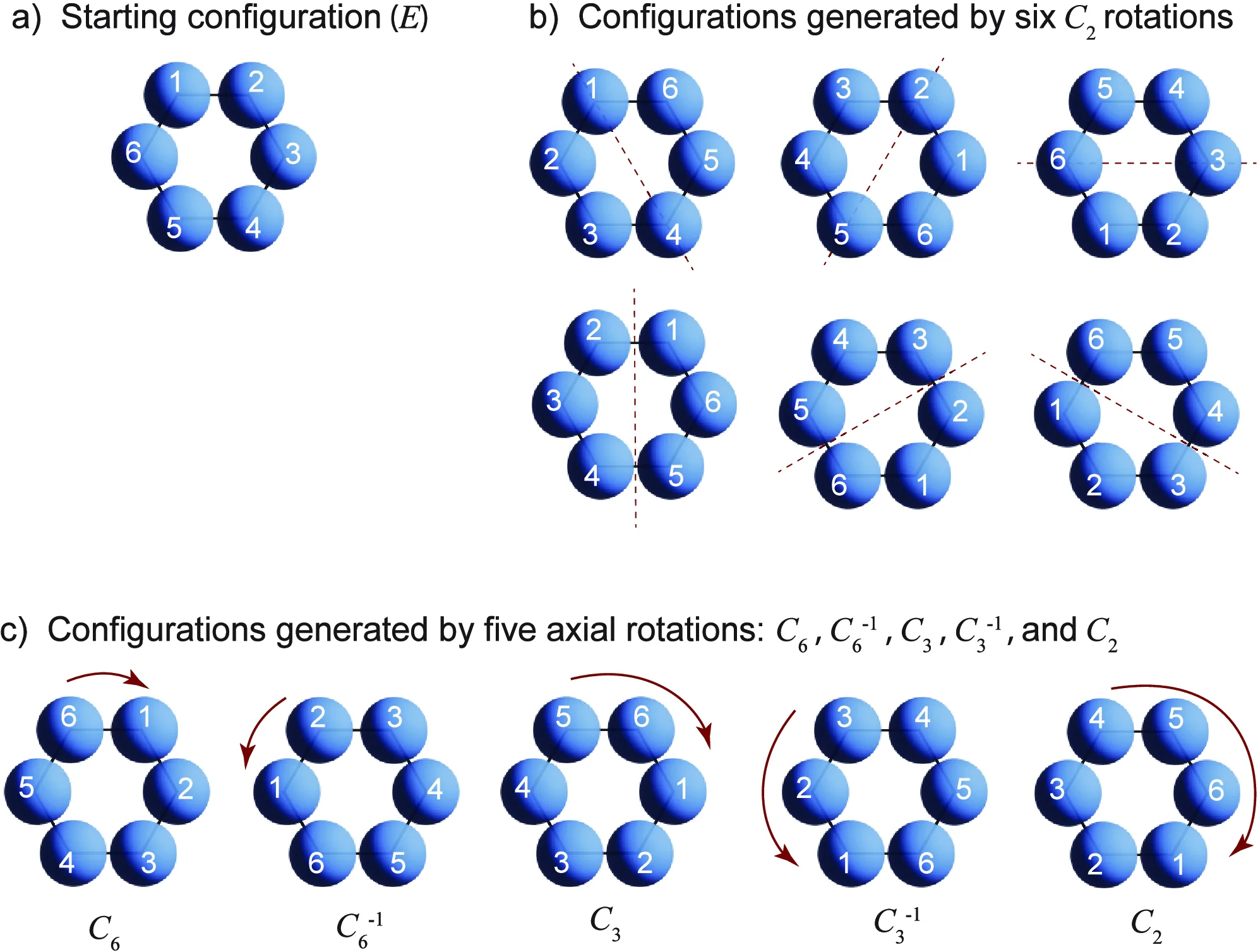
Use of all proper rotations of the *D*_6h_ point group is illustrated for generating the 12 identical configurations of a symmetric planar hexagon (e.g., benzene): (a) the arbitrary starting position; (b) illustrates all *C*_2_ rotations with dashed lines representing the axes, and (c) shows the results of all axial rotations.

## The Combinatorics Method

Using combinatorics does not require invoking rotations, and seeks σ as the number of identical configurations of a molecule in which the molecule does not have to be ‘broken’, i.e. preserving the relative position of atom labels that are declared in an arbitrary initial configuration. In short, one lists all possible (*N*!) labeling schemes of the *N* atoms and selects only those which can superimpose with the initial configuration. Although popularly used for diatomics and very simple molecules, combinatorics can become cumbersome to apply even when the number of atoms exceeds just 3 or 4.

Returning to methane (Figure 3), label the H atoms 1–4 in an arbitrary starting configuration (Figure 3 **a**). Fixing atom 1 to be the ‘top’ atom, there are three ways to distribute the remaining labels which preserve the relative label positions declared in Figure 3 **a**; notice there are three additional labeling options with 1 at the top position in Figure 3 **b** which do not superimpose with Figure 3 **a** and do not count. Next designate atom 2 to be the ‘top’, consider all six label permutations, and again notice that three cases are label-superimposable with the initial configuration and three are not (compare Figures 3 **a and** [Fig fig3] **b**). Repeating for remaining cases, all twelve possible configurations that are label-superimposable with the initial position are selected (Figure 3 **a**) with no mention of rotations. A second set of 12 superimposable configurations is also generated (Figure 3 **b**). To see an example, an efficient application of combinatorics to methane is given by Castellan.^24^

**Figure 3:**
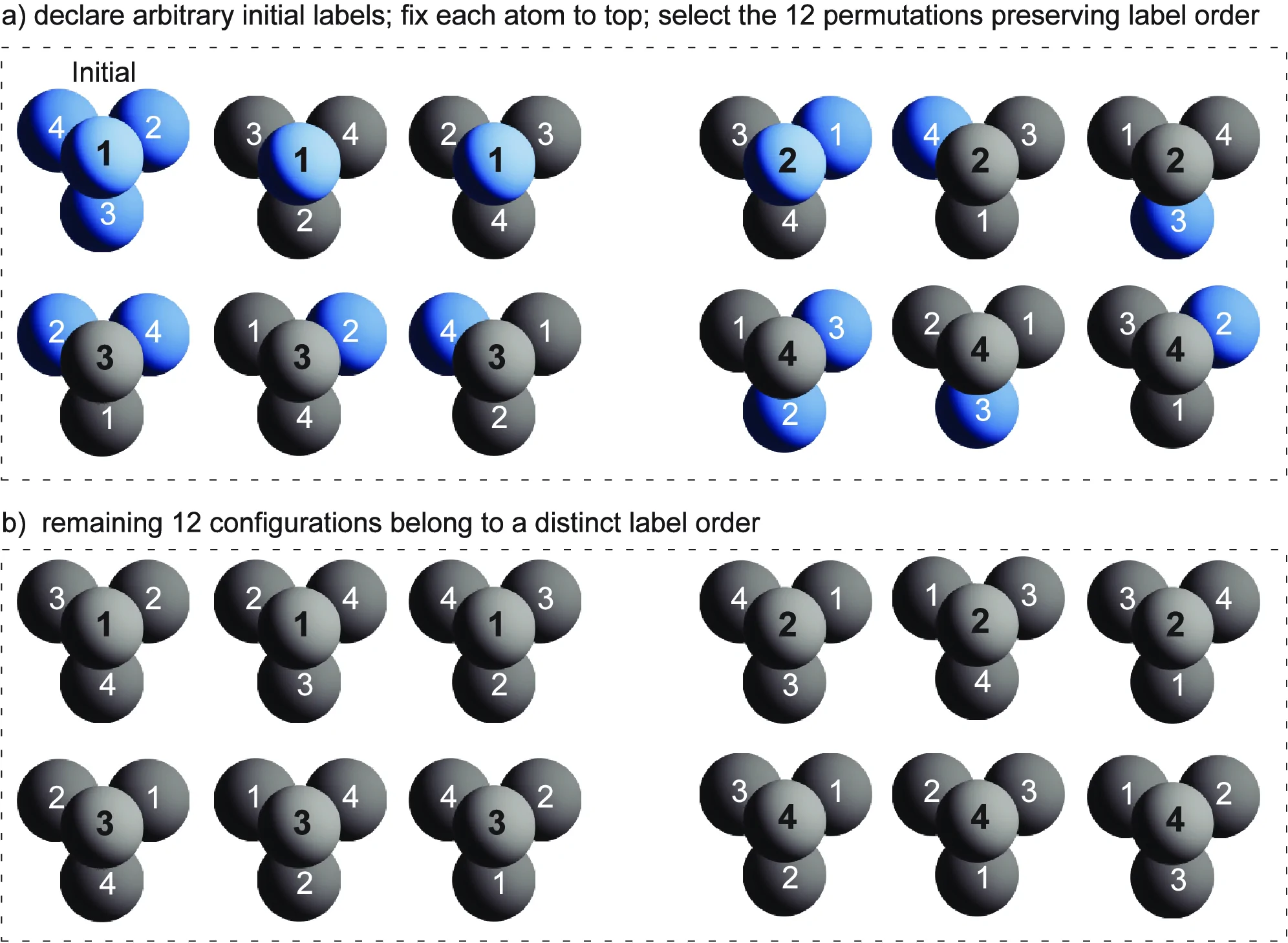
A combinatorics approach for generating the 12 identical configurations of CH_4_ is illustrated. There are *N*! = 24 permutations, but two distinct labeling schemes can be seen by contrasting panels (a) and (b), each of which has 12 configurations. The coloring scheme in (a) is the same as in Figure 1. Selecting allowed permutations (i.e., label superimposable with the starting configuration) can resemble rotation about the various axes, but rotations are not required. Using combinatorics often involves some rubric (e.g., setting each atom to be top) to help ensure all *N*! possibilities are considered.

A precaution in applying combinatorics is that, unless *N* is very small, one generally needs a systematic approach to help list and examine all *N*! options to filter out configurations that are not label-superimposable with the arbitrary starting configuration. In doing so, care should be taken to avoid using shortcuts that may confuse concepts. In other words, applying combinatorics involves first determining the number of nonsuperimposable label configurations, denoted here by *m*, where the example with methane showed that *m* = 2. The two distinct label configurations for methane are given in Figure 3 **a and** [Fig fig3] **b** respectively, differing on where atom 3 occurs relative to atoms 1 and 2. These nonsuperimposable labeling configurations can *sometimes* be thought of as ‘label enantiomers’,^4^ but only in simple cases such as methane where the distinct forms are obtained by reflection. Methane has *N*!=24 possible label configurations which can be divided into two subgroups, each with its own σ = 12 superimposable permutations as shown in Figure 3. Combinatorics requires that the *N*! possible permutations be divided by the number of nonsuperimposable labeling schemes *m* to obtain the symmetry number,

1 σ=N!/m

For example, the PCl_3_ trigonal pyramid also has two nonsuperimposable labeling schemes, so σ = 3!/2 = 3.

As *N* increases, the combinatorics approach becomes challenging in counting all *N*! possibilities and in determining *m*. Consider a perfect octahedron such as SF_6_ (symmetry group *O*_*h*_) which has *N*! = 720 possible label permutations of the F atoms. If we take σ = 24 as a given for the moment (Table 1), then one finds from Equation 1 that there are *m* = 30 nonsuperimposable labeling schemes. With a suitable rubric, one can count all 30 cases, but it is tedious and prone to mistakes to do so.

Combinatorics can still be invoked for SF_6_ though using a different system to help count and filter the 720 options, reiterating the precaution to avoid crutches that could be seen to overlap with other methods. As usual, declare an arbitrary initial configuration (Figure 4) and let one of the vertices be considered the ‘top’. If we fix the ‘top’ position to be label 1, then there are still 5!=120 possibilities to screen. However, fixing the top label to be 1 means that we can compare it to the initial state and eliminate all options in which the opposing atom is not a 6, leaving only 4!=24 cases for distributing the remaining labels to the four atoms in the plane equatorial to the 1–6 axis. Any of those four atoms can be labeled as a 2 but, once this is done, the remaining labels are constrained by the initial configuration. So for the case of fixing label 1 to the top, we have filtered 120 possibilities to only 4 that are superimposable with the initial label configuration. This procedure can be performed exactly 6 times in order to screen all 720 possibilities, so that $\sigma =\overset{6}{\underset{i=1}{\sum }}4=24$ .

**Figure 4:**
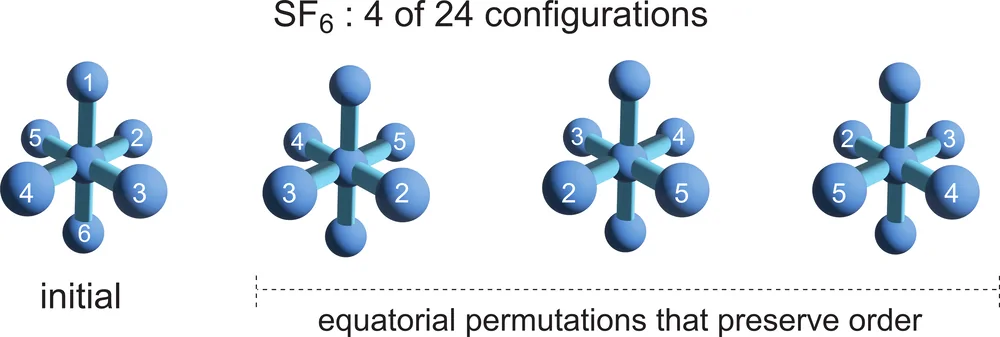
Four of the σ = 24 configurations of SF_6_. Counting these four configurations of SF_6_ can resemble performing successive rotations, but they are just the set of possibilities that preserve the label order defined in the initial position. Selecting each atom as the ‘top’ atom results in 24 configurations.

Recall that the use of combinatorics does not require invoking rotations. In the combinatorics procedure for SF_6_, it may be tempting to mention *C*_4_ rotations about a chosen SF bond as a shortcut to counting the options that preserve the label order in the permutations, but doing so could confuse this method with the proper rotations method.

## A Group Theoretical Method

The Schreier-Sims algorithm^3^ can be used to calculate the number of elements in a group of permutations and is implemented in the GAP (Groups, Algorithms, Processing) computer algebra software,^6^ which can be used to determine symmetry numbers of molecules, including accounting for internal motions. This section summarizes the Schreier-Sims algorithm that is used when GAP computes the order of groups (e.g., symmetry number), where usage and examples should be consulted in Gilson and Irikura,^5^ and a condensed method is given at the end of this section.

Let *G* be the rotational symmetry group of a molecule, and we wish to find the order of *G*. The Schreier-Sims algorithm finds a set of groups *G* ^(0)^...*G* ^(*n*–1)^, where *n* is the number of noncentral atoms in a molecule, *G* ^(0)^ = *G*, *G* ^(*i*)^⊆*G* ^(*i*–1)^, and *G* ^(*i*)^ holds the first *i* atoms fixed in space. Then find for each *G* ^(*i*)^, *i* > 0, a set *U* ^(*i*)^ which contains, for each position that the atom *i* can occupy in the group *G* ^(*i*–1)^, a member of *G* ^(*i*–1)^ that brings the atom *i* to the position that *G* ^(*i*)^ fixes it in space. The *U* ^(*i*)^ will then be used to generate σ.

An example with methane will help clarify these definitions (Figure 5). Label the H atoms of an arbitrary base position as *s* = (1, 2, 3, 4). We denote permutations by listing the locations of the atoms after applying the transformation. The central atom is not labeled since it is conserved in any rotation, so *n* = 4 and we wish to construct *U* ^(1)^, *U* ^(2)^, and *U* ^(3)^.

**Figure 5:**
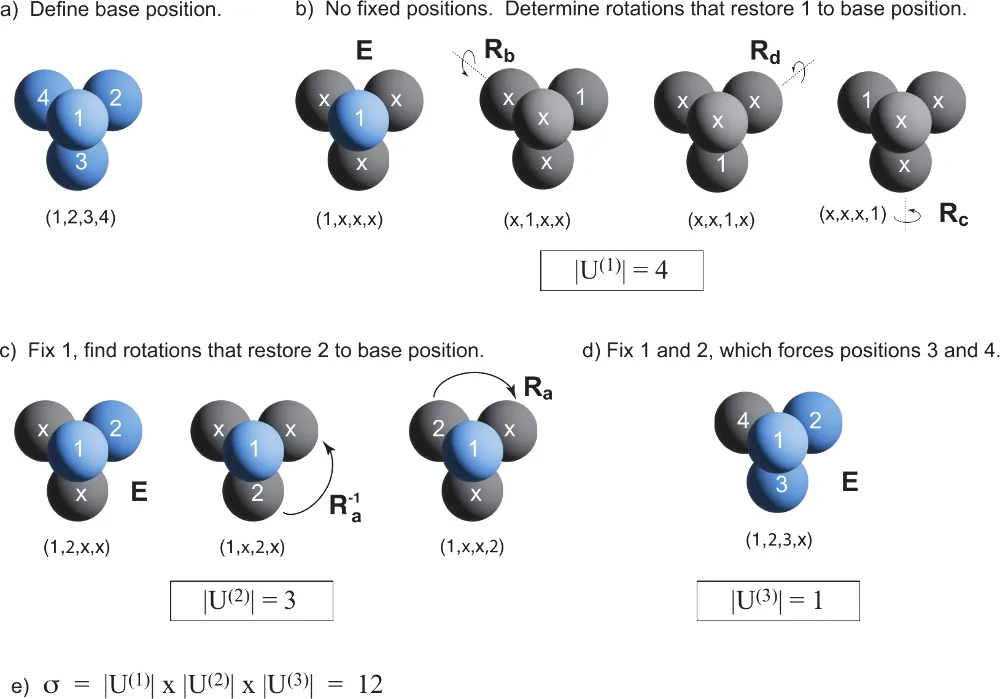
Application of the Schreier-Sims algorithm to methane (CH_4_). The algorithm can be thought of as an iteration of steps that involve fixing one additional atom in each pass.

The proper rotational subgroup of methane (Table 1) constitutes *G* = *G* ^(0)^. Denote clockwise rotations about the base positions 1, 2, 3, 4 as *R*_*a*_, *R*_*b*_, *R*_*c*_, *R*_*d*_ respectively. Then

2 $$\begin{array}{l} E\cdot s=\left(1, 2,3, 4\right),\\ R_{a}\cdot s=\left(1, 4,2, 3\right),\\ R_{b}\cdot s=\left(3, 2,4, 1\right),\\ R_{c}\cdot s=\left(4, 1,3, 2\right),\\ R_{d}\cdot s=\left(2, 3,1, 4\right), \end{array}$$

where *R*_a_, *R*_b_, *R*_c_, *R*_d_ are recognized as the four *C*_3_ rotations of the proper rotational subgroup.

Starting with *i* = 1, a rotational subgroup *G* ^(1)^ that fixes atom 1 to the ’top’ position of the base configuration consists of the *C*_3_ and *C*_3_^–1^ operations about atom 1 as well as identity (*E*): *G* ^(1)^ = {*E*, *R*_*a*_,*R*_*a*_^–1^} . Next consider the possible positions of atom 1 and then find the set *U* ^(1)^ of rotations that bring atom 1 to its base position (Figure 4 **B**). The four starting positions that atom 1 can occupy are:

3 $$\begin{array}{c} \left(1,?,?,?\right),\\ \left(?,1,?,?\right),\\ \left(?,?,1,?\right),\\ \left(?,?,?,1\right), \end{array}$$

where ’?’ indicates that the identity of that element is not needed. For the first case, atom 1 is already in its original position so the corresponding member of *U* ^(1)^ is *E*, although either *R*_*a*_ or *R*_*a*_^–1^ could be chosen. Redundant choices can occur in this step and only one need be chosen. For the second case, atom 1 can be returned to its original position by applying either *R*_*c*_^–1^ or *R*_*b*_, where we arbitrarily choose *R*_*b*_. For the third position we can choose *R*_*d*_, and for the fourth position, *R*_*c*_, (Figure 5 **B**), leading to:

4 $$U^{\left(1\right)}=\left\{E,R_{b},R_{d},R_{c}\right\}$$

where *U* ^(1)^ could be comprised of other rotations, but must have four elements. Every element of *U* ^(1)^ is a member of *G* ^(0)^. We can use *U* ^(1)^ to bring atom 1 to the top from any arbitrary starting position. Recall that any member of the *G* ^(1)^ rotational subgroup will preserve atom 1 in its position. Next consider a subgroup *G* ^(2)^ that fixes positions 1 and 2, and therefore only consists of *E*. Identify all possible positions of atom 2 and corresponding rotations that bring atom 2 to its base position (Figure 5 **C**):

5 $$\begin{array}{c} \left(1, 2,?,?\right),\\ \left(1,?,2,?\right),\\ \left(1,?,?,2\right). \end{array}$$

In the first case, atom 2 is already in its base position so the corresponding element of *U* ^(2)^ is *E*. For the second possibility, we must return atom 2 to its base position without moving atom 1, which can be accomplished with *R*_*a*_^–1^. For the third case, choose *R*_*a*_ to bring atom 2 to the base position, yielding

6 $$U^{\left(2\right)}=\left\{E,R_{a}^{-1},R_{a}\right\}$$

Notice that *U* ^(2)^ consists of members of *G* ^(1)^. The final stage is to fix 1 and 2 to their base positions and consider all possible starting positions of atom 3 in order to find *U* ^(3)^. There may appear to be two possibilities,

(1,2,3,4),and(1, 2,3, 4),

however the second case is impossible because it violates the relative order in the base position and would break the molecule. Since the only option is that in which 3 is already in its base position, then *U* ^(3)^ consists solely of *E* (Figure 5 **D**), which is a member of *G* ^(2)^. Connections to *G* ^(*i*)^ have been pointed out, however we can trust Schreier-Sims to hold and could have focused solely on the *U* ^(*i*)^. In order to find the symmetry number, recognize that any arbitrary state can be returned to the initial configuration by moving atoms to their initial positions one at a time using *U* ^(1)^...*U* ^(*n*–1)^. Therefore, the number of unique rotations must be given by the number of possible choices of members from each *U* ^(1)^...*U* ^(*n*–1)^, which is the product of the sizes of those sets

7 $$\left|G\right|=\left|U^{\left(1\right)}\right|\cdot \left|U^{\left(2\right)}\right|\cdot \left|U^{\left(3\right)}\right|=4\cdot 3\cdot 1=12$$

It can be appreciated now that some informal approaches source the same numbers (4·3 = 12) as the group theory algorithm, but without the associated justifications.

The Schreier-Sims algorithm can appear daunting, but the GAP software handles it for users, while a fast and intuitive version is applicable to rigid molecules and uses only two iterations, illustrated next. This condensed Schreier-Sims method focuses only on the necessary parts (recall we do not need to enumerate the *U* ^(i)^ sets), and requires only making appropriate choices of the first two atom labels 1 and 2.

First declare a starting configuration of only labels 1 and 2 (Figure 5 **A**), *where atoms labeled 1 and 2 do not share a rotational axis*. Next count all possible locations of atom 1 that can be returned to the starting position by a rotation in the symmetry group (Figure 5 **B**), including the starting position. Finally, fix atom 1 to its starting position and count all rotationally allowed positions of atom 2 (Figure 5 **C**), again including the starting position. The symmetry number is the product of the orders of these two steps, termed here a *condensed Schreier-Sims product* (Table 1).

In other words, using two linearly independent rotational axes is sufficient to constrain σ. Additional examples:

- *XY*_*4*_ *tetrahedron*. No two Y atoms share a common rotational axis, so declare an arbitrary initial configuration and then observe that atom 1 has four possible positions that can be returned to the initial position. Fix atom 1 and observe that atom 2 has only three possible positions that can be returned to the initial place by rotation, arriving at the condensed Schreier-Sims product σ = 4·3 = 12.
- *XY*_*6*_ *octahedron*. Recall that atoms 1 and 2 must not share a rotational axis in this fast method, so any atom can be set to 1, but the opposing Y atom must not be labeled 2. So any one of the remaining four atoms that are equatorial to atom 1 must be labeled as atom 2. Atom 1 has six possible locations that can be returned to a starting position rotationally. After fixing atom 1, atom 2 has four possible positions related by rotation. Then σ = 6·4 = 24.
- *XY*_*4*_ *square plane*. Let the Y atoms be labeled 1–4, where the placement of label 1 is arbitrary, but label 2 must be placed on a Y atom adjacent to atom 1 to avoid sharing a common rotational axis. Atom 1 has four possible locations that can be returned to an arbitrary initial position by rotations. Fixing atom 1 to its initial location, atom 2 can only have two positions. Then σ = 4·2 = 8.

This condensed method only requires that atoms 1 and 2 are placed on linearly independent rotational axes. As assurance, Schreier-Sims is general for any labeling scheme, but could then involve more than two iterations. For example, if the initial F atom numbering in XeF_4_ was (1,3,2,4), thereby placing atoms 1 and 2 on the same rotational axis, the Schreier-Sims procedure would converge in three iterations (4·1·2 = 8 for atoms 1, 2, and 3 respectively), which still simplifies to a two-term product (4·2).

The Schreier-Sims method is un-necessary for simple diatomics, and can also become complex as *N* increases. Consider uranocene (U­(C_8_H_8_)_2_), which belongs to the symmetry group *D*_*8h*_ in its eclipsed configuration of two sandwiched 8-membered rings.^49^ It would take some practice to recognize that the condensed Schreier-Sims product is 16·1. Instead, the order of the proper rotational subgroup can be quickly found as 16 from references,^48^ or one can use the rule that σ­(*D*_*nh*_) = 2*n* for finite *n* ≥ 2.

## Conclusion and Recommendations

The symmetry number concept serves both current inquiry and foundations in physical chemistry, where source materials superbly present the complex and potent field of statistical chemical thermodynamics to diverse audiences. Based on a meta-analysis of print and online sources, the IUPAC definition of σ, and a survey of three methods to determine σ, it is supported here that exposition of the classical symmetry number be expanded.

The IUPAC definition of σ should be more widely included, and viewed as essential in any setting that explores the application of σ to the rotational properties of molecules (e.g., Table 1). Even if the symmetry number concept is presented minimally for diatomics, e.g. σ = 2 (*homonuclear*) or 1 (*heteronuclear*), the inclusion of the IUPAC definition gives a more complete representation of the σ concept.

Next, a method should be identified for determining σ, being careful to avoid informal methods and to not treat σ as obvious, while providing sufficient exposition on the chosen method to help readers tackle new cases. Three formal methods were reviewed here (order of proper rotational subgroup, combinatorics, Schreier-Sims), which all have various merits and challenges.

Although it is not in the scope of this Viewpoint to explore specific class modalities and pedagogies, increased adoption of the method of using the order of the proper rotational subgroup is recommended due to its natural connection to the IUPAC definition and its relative ease in many cases. More advanced materials, or those that consider computation, may wish to further incorporate combinatorics or the Schreier-Sims procedure (using GAP and/or the condensed algorithm) as supplemental topics.

These recommendations aim to promote increased adoption of standards and associated methods, independent of specific venues or teaching modalities.

## References

[1] International Union of Pure and Applied Chemistry (IUPAC) . Symmetry Number. 10.1351/goldbook.S06214.

[2] Mayer J. E., Brunauer S., Mayer M. G. (1933). The Entropy
of Polyatomic Molecules
and the Symmetry Number. J. Am. Chem. Soc..

[3] SIMS, C. C. Computational Methods in the Study of Permutation groups† †This Research Was Supported in Part by the National Science Foundation. In Computational Problems in Abstract Algebra, LEECH, J. , Ed.; Pergamon, 1970; pp 169–183. 10.1016/B978-0-08-012975-4.50020-5.

[4] Ercolani G., Piguet C., Borkovec M., Hamacek J. (2007). Symmetry Numbers and
Statistical Factors in Self-Assembly and Multivalency. J. Phys. Chem. B.

[5] Gilson M. K., Irikura K. K. (2010). Symmetry Numbers
for Rigid, Flexible, and Fluxional
Molecules: Theory and Applications. J. Phys.
Chem. B.

[6] GAP – Groups, Algorithms, and Programming, Version 4.15.1, The GAP Group, 2025.

[7] Pollak E., Pechukas P. (1978). Symmetry Numbers, Not
Statistical Factors, Should Be
Used in Absolute Rate Theory and in Broensted Relations. J. Am. Chem. Soc..

[8] Fernández-Ramos A., Ellingson B. A., Meana-Pañeda R., Marques J. M. C., Truhlar D. G. (2007). Symmetry
Numbers and Chemical Reaction Rates. Theor.
Chem. Acc..

[9] Prats H. (2025). ZacrosTools:
A Python Library for Automated Preparation, Analysis, and Visualization
of Kinetic Monte Carlo Simulations with Zacros. J. Phys. Chem. A.

[10] Chen J., Tang T. (2024). Density Functional
Theory-Based Kinetic Modeling of Reactions of
Hydrogen Isotopes (H2, D2, T2) and Carbonaceous Gases (CO2, CO, CH4)
on the ZrCo(110) Surface. J. Phys. Chem. C.

[11] Kubečka J., Besel V., Kurtén T., Myllys N., Vehkamäki H. (2019). Configurational
Sampling of Noncovalent (Atmospheric) Molecular Clusters: Sulfuric
Acid and Guanidine. J. Phys. Chem. A.

[12] Pitzer K. S. (1939). The Symmetry
Number and Thermodynamic Functions for Molecules Having Double Minimum
Vibrations. J. Chem. Phys..

[13] Estrada E., Avnir D. (2003). Continuous Symmetry
Numbers and Entropy. J.
Am. Chem. Soc..

[14] Pitzer, K. S. ; Gwinn, W. D. Energy Levels and Thermodynamic Functions for Molecules with Internal Rotation I. Rigid Frame and Attached Tops. J. Chem. Phys. 1942, 10, 428 10.1063/1.1723744

[15] Lewis, G. N. ; Randall, M. ; Pitzer, K. S. ; Brewer, L. Thermodynamics, 2nd ed.; McGraw-Hill: New York, 1961.

[16] Di
Stefano S., Ercolani G. (2016). Catenation Equilibria Between Ring
Oligomers and Their Relation to Effective Molarities: Models From
Theories and Simulations. Macromol. Theory Simul..

[17] McQuarrie, D. A. . Statistical Mechanics; Harper&s Chemistry Series; Harper & Row: New York, NY, 1973.

[18] Alberty, R. A. ; Silbey, R. J. Physical Chemistry, Second; John Wiley & Sons, Inc., 1997.

[19] Atkins, P. ; de Paula, J. ; Keeler, J. Physical Chemistry, 11th ed.; Oxford University press, 2018.

[20] Ball, D. W. ; Baer, T. Physical Chemistry, 2nd ed.; Wadsworth Cengage Learning: Stamford, CT, 2015.

[21] Barrow, G. M. Physical Chemistry, 4th ed.; McGraw-Hill Inc., 1979.

[22] Barrow, G. M. Physical Chemistry, 5th ed.; McGraw-Hill Inc., 1988.

[23] Blinder, S. M. Advanced Physical Chemistry, The Macmillan Company: London, 1969.

[24] Castellan, G. A. Physical Chemistry, Third Edition, 3rd ed.; Addison-Wesley: Reading, MA, 1983.

[25] Daniels, F. ; Alberty, R. A. Physical Chemistry, Third; John Wiley & Sons, Inc., 1966.

[26] Davis, J. C. Advanced Physical Chemistry, The Ronald Press Company: New York, NY.

[27] Engel, T. ; Reid, P. Physical Chemistry, 3rd ed.; Pearson, 2013.

[28] Eyring, H. ; Walter, J. ; Kimball, G. E. Quantum Chemistry, John Wiley & Sons, Inc.: New York, NY, 1944.

[29] Fowler, R. H. ; Guggenheim, E. A. Statistical Thermodynamics, Cambridge University Press: London, 1939.

[30] Grossman, L. M. Thermodynamics and Statistical Mechanics, McGraw-Hill Inc., 1969.

[31] Hecht, C. E. Statistical Thermodynamics and Kinetic Theory, W. H. Freeman and Co.: New York, 1990.

[32] Hill, T. Statistical Mechanics: Principles and Selected Applications, Dover Publications, 1987.

[33] Hirschfelder, J. O. ; Curtiss, C. F. ; Bird, R. B. Molecular Theory of Gases and Liquids, John Wiley & Sons, Inc.: New York, 1954.

[34] Laidler, K. J. ; Meiser, J. H. ; Sanctuary, B. C. Physical Chemistry, Houghton Mifflin Company.

[35] Levine, I. N. Physical Chemistry, 6th ed.; McGraw-Hill: New York, NY, 2009.

[36] McQuarrie, D. A. ; Simon, J. D. Physical Chemistry: A Molecular Approach, University Science Books, 1997.

[37] Moore, W. J. Physical Chemistry, 5th ed.; Longman Scientific & Technical: London, 1972.

[38] Reif, F. Fundamentals of Statistical and Thermal Physics, McGraw-Hill, Inc., 1965.

[39] Rushbrooke, G. S. Introduction to Statistical Mechanics, Clarendon Press: Oxford, 1962.

[40] Silbey, R. J. ; Alberty, R. A. ; Bawendi, M. G. Physical Chemistry, 4th ed.; John Wiley & Sons, Inc., 2004.

[41] Sonntag, R. E. ; Van Wylen, G. J. Fundamentals of Statistical Thermodynamics, John Wiley & Sons: New York, NY, 1966.

[42] Steinfeld, J. I. Molecules and Radiation, 2nd Ed.; The MIT Press: Cambridge, MA, 1985.

[43] Tien, C. L. ; Lienhard, J. H. Statistical Thermodynamics, Hemisphere Publishing Corporation, 1979.

[44] Tolman, R. C. The Principles of Statistical Mechanics, Oxford University Press: London, 1959.

[45] Rotational Partition Functions Contain a Symmetry Number. https://chem.libretexts.org/@go/page/51129 (accessed 2026–02–18).

[46] The Rotational Partition Function of A Diatomic Ideal Gas. https://chem.libretexts.org/@go/page/152807 (accessed 2026–02–18).

[47] Rotational Partition Functions of Polyatomic Molecules Depend on the S phar of the Molecule. https://chem.libretexts.org/@go/page/13698(accessed 2026–02–18).

[48] Cotton, F. A. Chemical Applications of Group Theory, 2nd ed.; John Wiley & Sons, Inc., 1971.

[49] Seyferth D. (2004). Uranocene.
The First Member of a New Class of Organometallic Derivatives of the
f Elements. Organometallics.

